# Supplementary material for: Serine/Threonine Phosphatases in LTP: Two B or Not to Be the Protein Synthesis Blocker-Induced Impairment of Early Phase
Source: Int J Mol Sci. 2021 May 4;22(9):4857. doi: 10.3390/ijms22094857 (PMC8125358; doi:10.3390/ijms22094857)
Supplement: Supplementary file 1 [file ijms-22-04857-s001.zip › ijms-1195223-SI.pdf]

## Supplemental Data

**Serine/threonine phosphatases in LTP: Two B or not to be the protein synthesis blocker-induced impairment of early phase**

Alexander V. Maltsev, Natalia V. Bal, Pavel M. Balaban.

Institute of Higher Nervous Activity and Neurophysiology, Russian Academy of Sciences, 117485, Butlerova 5A, Moscow, Russia

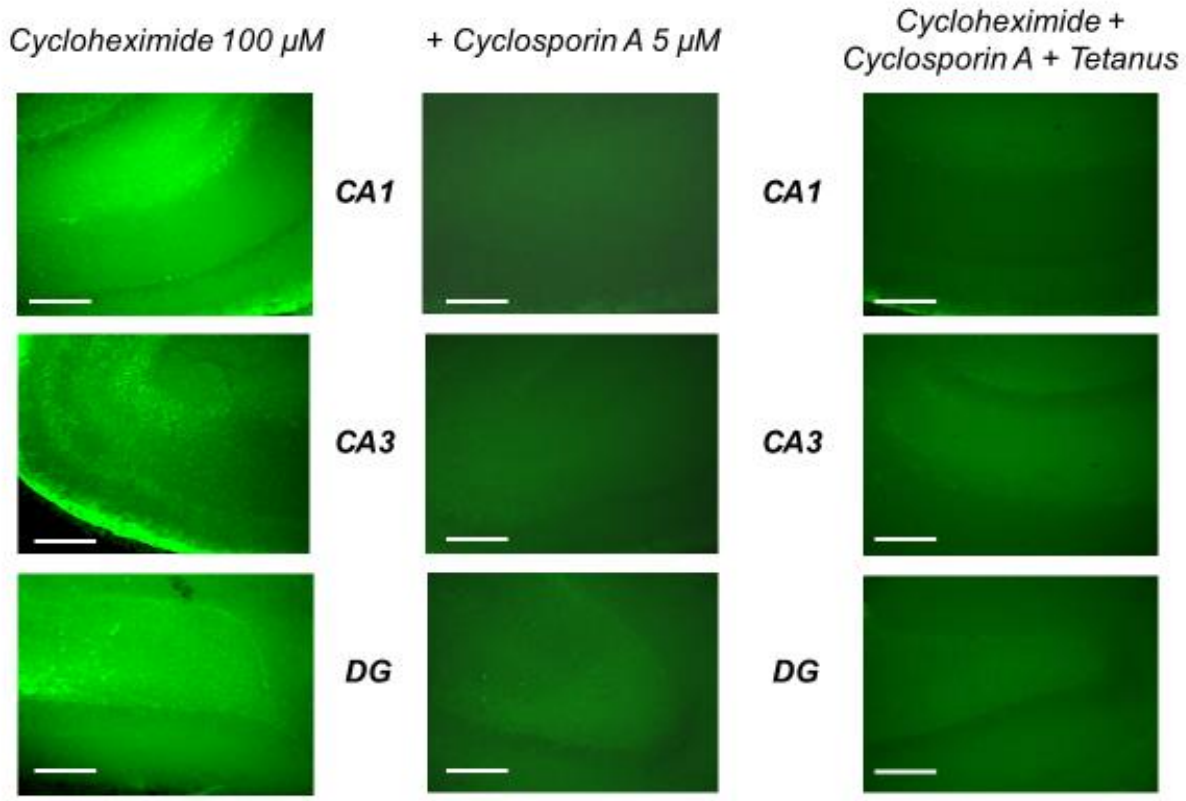


**Suppl. Fig. 1.** Cycloheximide-induced NO production in CA1, CA3 and DG in the hippocampus (left panels) and its prevention in the presence of cyclosporin A without (middle panels) and 30 min after tetanic induction (right panels). Scale bars 400 μm.

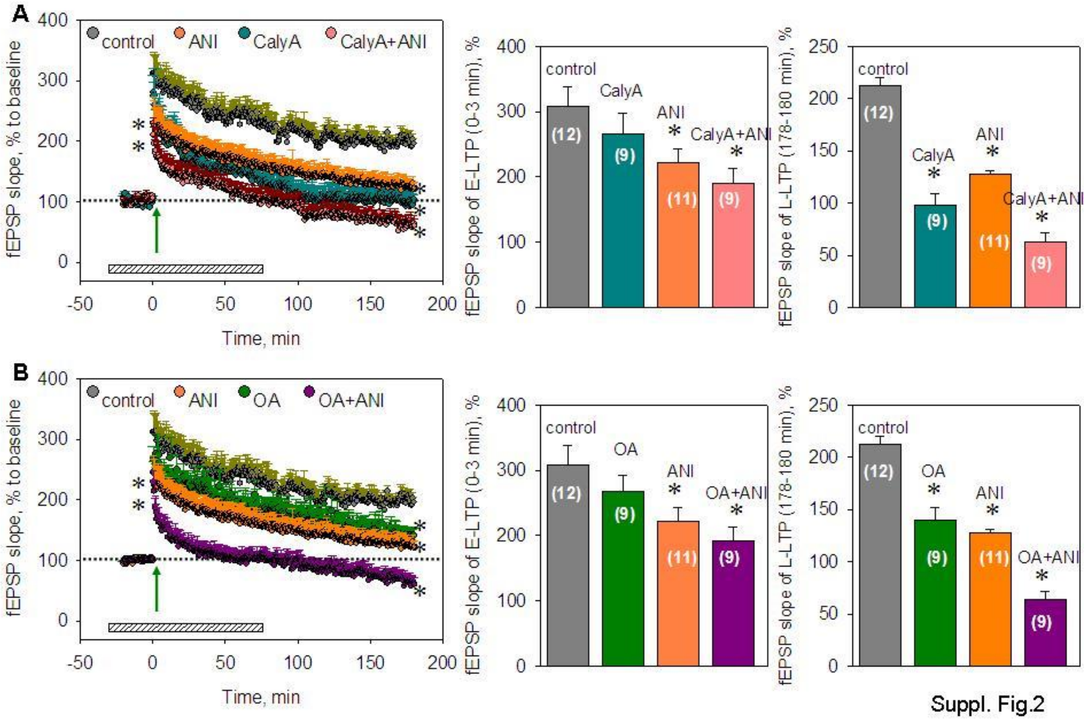


**Suppl. Fig. 2.** PP1/PP2A inhibitors do not prevent the PSB-induced E-LTP impairment. Pretreatment of hippocampal slices in the presence of 50 nM calyculin A, CalyA (A) or 100 nM Okadaic acid, OA (B) have no influence on the 25 μM anisomycin-impaired LTP. Number of experiments is indicated in parentheses.* denotes $p < 0.05$ *vs.* the control.

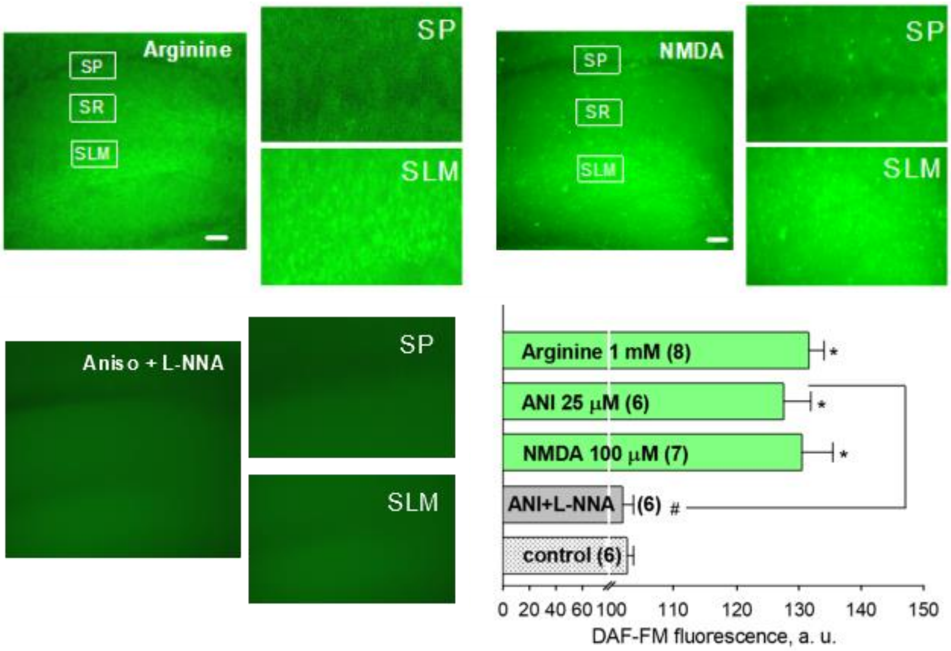


**Suppl. Fig. 3. NO synthesis produced by the endogenous NO donor Arginine and stimulation of NMDA-receptors, as well preventing of anisomycin-induced NO synthesis in the presence of the NO synthase blocker L-NNA (100 μM).** SP – *stratum pyramidale*, SR – *stratum radiatum*, SLM – *stratum lacunosum-moleculare*. Number of independent slices are presented in parentheses, * - $p < 0.05$ *vs.* control, # - $p < 0.05$ for comparison of groups between themselves.
